# Fructose-mediated AGE-RAGE axis: approaches for mild modulation

**DOI:** 10.3389/fnut.2024.1500375

**Published:** 2024-12-04

**Authors:** Halyna Semchyshyn

**Affiliations:** Department of Biochemistry and Biotechnology, Vasyl Stefanyk Precarpathian National University, Ivano-Frankivsk, Ukraine

**Keywords:** fructose, nonenzymatic processes, reactive species, glycation products, receptor for advanced glycation end products, diet optimization, sulforaphane, physical activity

## Abstract

Fructose is a valuable and healthy nutrient when consumed at normal levels (≤50 g/day). However, long-term consumption of excessive fructose and elevated endogenous production can have detrimental health impacts. Fructose-initiated nonenzymatic glycation (fructation) is considered as one of the most likely mechanisms leading to the generation of reactive species and the propagation of nonenzymatic processes. In the later stages of glycation, poorly degraded advanced glycation products (AGEs) are irreversibly produced and accumulated in the organism in an age- and disease-dependent manner. Fructose, along with various glycation products—especially AGEs—are present in relatively high concentrations in our daily diet. Both endogenous and exogenous AGEs exhibit a wide range of biological effects, mechanisms of which can be associated with following: (1) AGEs are efficient sources of reactive species *in vivo*, and therefore can propagate nonenzymatic vicious cycles and amplify glycation; and (2) AGEs contribute to upregulation of the specific receptor for AGEs (RAGE), amplifying RAGE-mediated signaling related to inflammation, metabolic disorders, chronic diseases, and aging. Therefore, downregulation of the AGE-RAGE axis appears to be a promising approach for attenuating disease conditions associated with RAGE-mediated inflammation. Importantly, RAGE is not specific only to AGEs; it can bind multiple ligands, initiating a complex RAGE signaling network that is not fully understood. Maintaining an appropriate balance between various RAGE isoforms with different functions is also crucial. In this context, mild approaches related to lifestyle—such as diet optimization, consuming functional foods, intake of probiotics, and regular moderate physical activity—are valuable due to their beneficial effects and their ability to mildly modulate the fructose-mediated AGE-RAGE axis.

## Introduction

Vital processes in living organisms are closely associated with reactive species, which represent a very diverse group from the point of view of both their chemistry and biological impact. These include molecules, atoms, and ions. Some of them belong to a family known as highly reactive free radicals. In aerobic organisms, many reactive species derive from molecular oxygen, some of them are nitrogen- and sulfur-containing, or have other chemical features, for example reactive carbonyl group(s). Although reactive species can be produced endogenously, for instance, as side products of enzymatic metabolism, they are often involved in nonenzymatic reactions (1–4). Unlike enzymatic reactions, which are precisely controlled by respective enzymes, nonenzymatic processes are rather unspecific and occur spontaneously. Generally, nonenzymatic events and reactive species are well-known for playing a detrimental role associated with health risks. On the other hand, being an important part of immune response, regulators of gene expression, and cellular signaling messengers, reactive species at low concentrations can have beneficial effects (5–9).

Historically, most studies in the field of reactive species and nonenzymatic processes in living organisms were focused on free radical oxidation and glycation, related to reactive oxygen species (ROS) and reactive carbonyl species (RCS) in particular. Increased levels of ROS and RCS lead to development of oxidative and carbonyl stresses, respectively. Obviously, both nonenzymatic processes mentioned above are closely linked to each other—oxidation reactions and ROS are found to be involved in certain stages of glycation, at the same time glycation itself is a source of ROS and ROS-mediated reactions (10–12). To reflect the combinational impact of nonenzymatic oxidation and glycation, the term “glycoxidation” has been introduced and is commonly used (13, 14). In addition, another nonenzymatic process such as ROS-induced lipid peroxidation results in a variety of RCS and respective glycation products, demonstrating an interplay between oxidative and carbonyl stresses. Glycoxidation leads to irreversible modifications of biomolecules and damages to cellular constituents with the consequent formation of poorly degraded compounds, collectively named advanced glycation end products (AGEs). Elevated levels of AGEs initiate activation of the specific receptor for AGEs (RAGE), mediating and amplifying inflammatory events related to metabolic disorders, chronic diseases, and aging (4, 15, 16). In this regard, inhibiting the AGE-RAGE axis is considered as a logical target in pathological conditions.

Reducing carbohydrates are major contributors to glycation process under physiological conditions. Among other monosaccharides, fructose has been found to be one of the most potent glycation agents *in vivo* (17–20). Numerous studies have demonstrated adverse metabolic changes in humans and animals as deleterious side effects of long-term intake of excessive fructose. These effects could be attributed to fructose-mediated glycation, increased levels of RCS and ROS, AGE accumulation, and subsequent activation of RAGE (18, 21–26).

Importantly, there is compelling evidence that short-term application of moderate doses of fructose can have beneficial defensive effects under certain pathophysiological conditions, particularly those associated with oxidative/carbonyl stress (21, 23, 27–30). Therefore, strategies such as diet optimization—limiting added sugars, and fructose in particular—could effectively prevent overactivation of RAGE signaling, and thereby mitigate the detrimental impacts of fructose. Other mild approaches may also offer moderate modulation of the fructose-mediated AGE-RAGE axis.

## Fructose-initiated glycation and endogenous/exogenous glycotoxins

**Figure 1:**
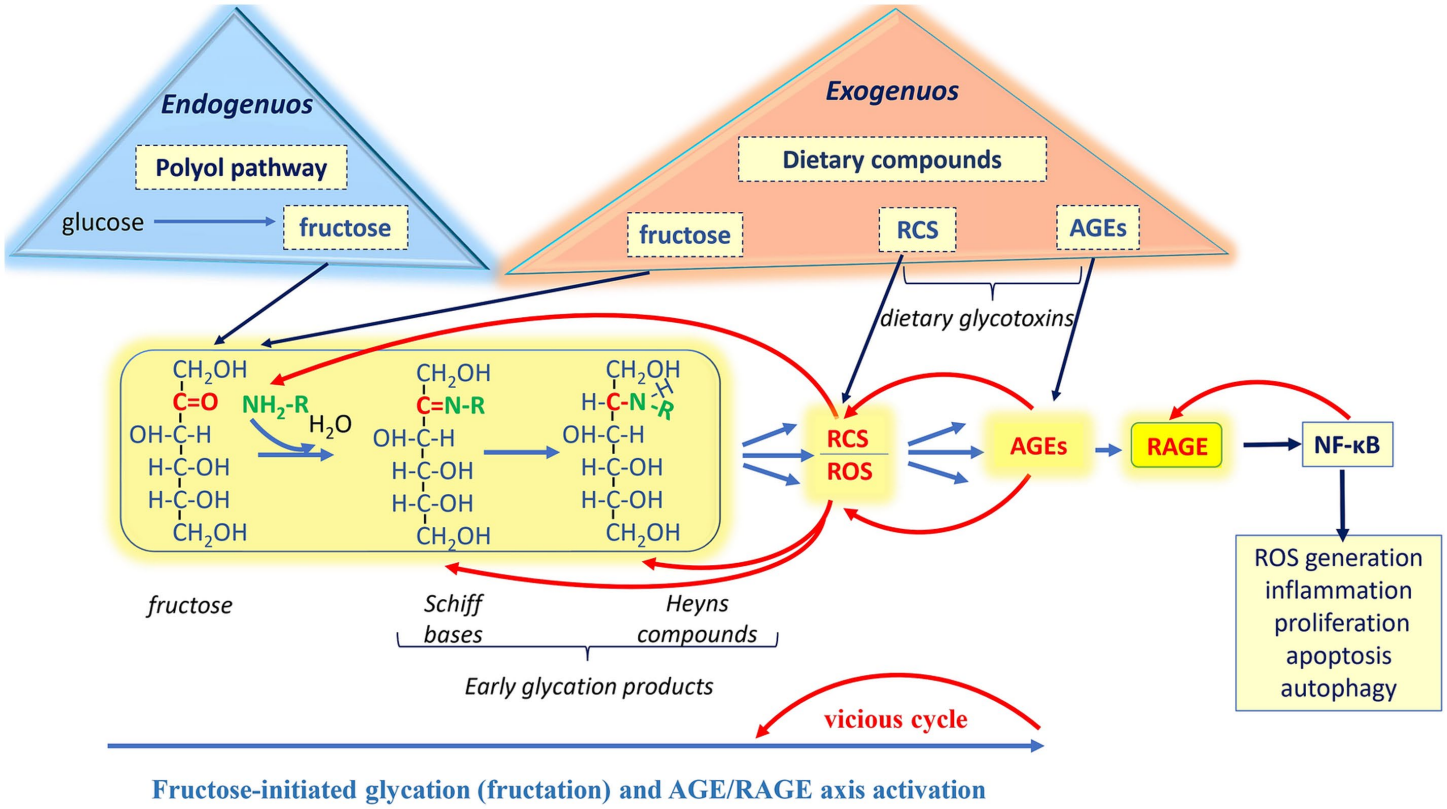
Involvement of exogenously and endogenously derived fructose in the generation of Reactive carbonyl species (RCS), Reactive oxygen species (ROS), and Advanced glycation end products (AGEs), followed by the activation of the Receptor for advanced glycation end products (RAGE). Nonenzymatic reactions: Fructose-initiated glycation. The initial step of the glycation is a covalent interaction between the electrophilic carbonyl group of open-chain fructose and the nucleophilic functional group of a biomolecule. This interaction produces a diverse group of early glycation products (Schiff bases and Heyns products). Schiff bases can undergo further isomerization to form more stable Heyns adducts. The fructose moiety of the Heyns products can undergo enolization, followed by dehydration, oxidation, and/or fragmentation reactions, consequently producing a variety of reactive species. In the advanced stages of glycation, early products and reactive species further undergo complex of irreversible nonenzymatic reactions, ultimately producing a variety of AGEs. Enzymatic reactions: Under hyperglycemia, in the insulin-insensitive tissues, polyol pathway is responsible for converting glucose to fructose, which in turn may initiate glycation. Dietary fructose and glycation products: Most foods and beverages contain added fructose and dietary glycotoxins (RCS and AGEs) in substantive amounts as compared to their endogenous sources. Both exogenously and endogenously derived AGEs bind to the receptor RAGE, which is involved in a variety of biological processes. Stimulation of RAGE leads to activation of the transcription nuclear factor kappa-B (NF-κB), which is associated with an enhancement of prooxidative and proinflammatory responses. In turn, NF-κB increases RAGE expression, which again stimulates NF-κB, forming a vicious cycle. Upregulation of RAGE is associated with various normal physiological processes and pathologies.

The initial step of fructose-initiated glycation (Figure 1) is a covalent interaction between electrophilic carbonyl group of open-chain fructose and nucleophilic functional group of a biomolecule. This interaction produces a diverse group of early glycation products (Schiff bases and Heyns products). Schiff bases are relatively unstable compounds that can be subjected to further isomerization (Heyns rearrangement) and formation of more stable Heyns adducts. Amadori products derived from glucose are also belong to early glycation products. The fructose moiety of the Heyns products can undergo enolization followed by dehydration, oxidation, and/or fragmentation reactions, consequently producing a variety of reactive species (10, 13, 31).

**Figure 2:**
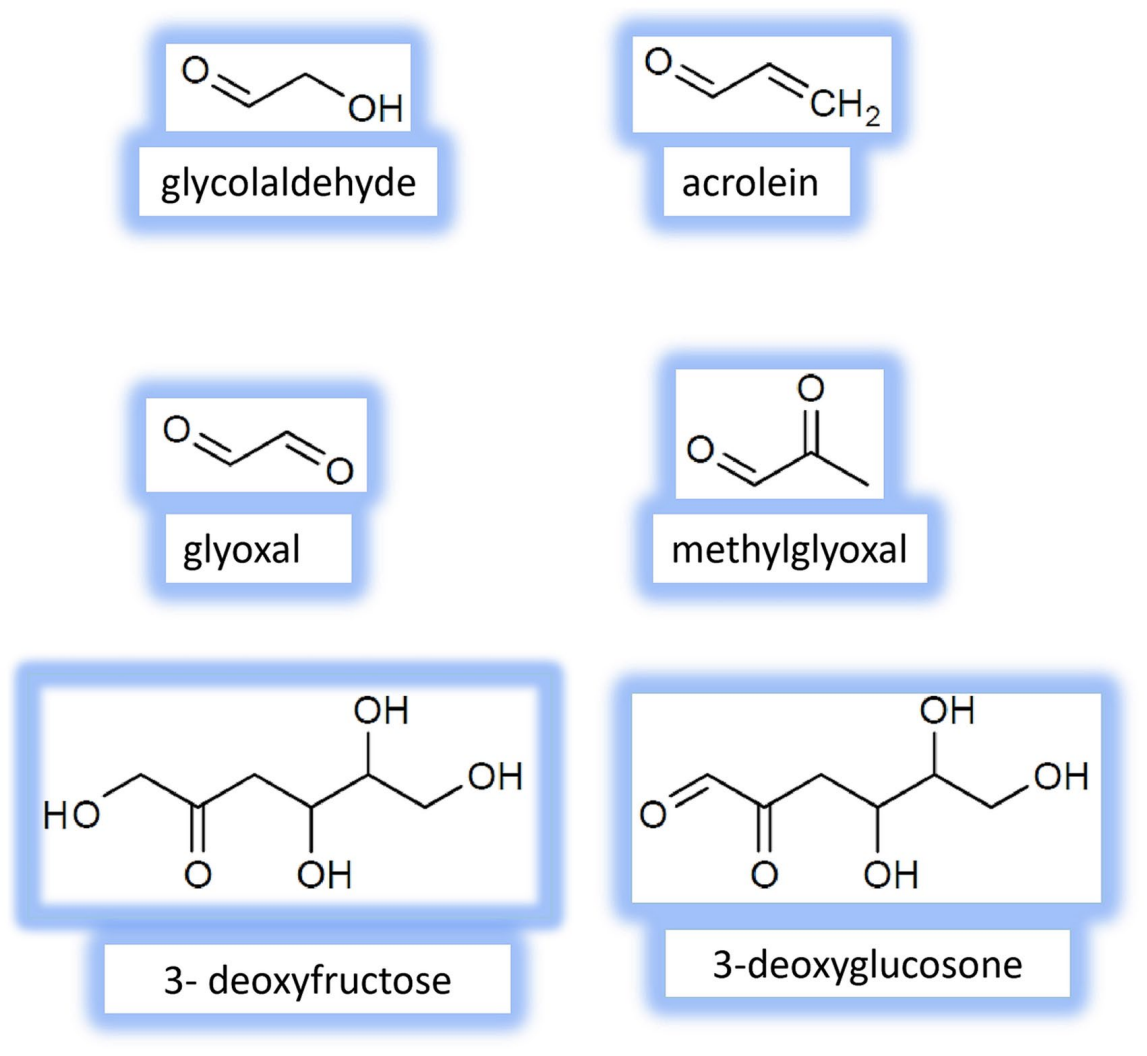
Chemical structure of the main fructose-derived Reactive carbonyl species (RCS) identified *in vivo.*

Besides glycation, other nonenzymatic reactions contribute to the formation of reactive species. They are autoxidation of glucose, fructose and other monosaccharides (Wolff pathway), autoxidation of Amadori and Heyns compounds (Hodge pathway), and oxidative fragmentation of Schiff bases (Namiki pathway) (2, 11, 14, 32, 33). Some fructose-derived RCS have been identified *in vivo* (Figure 2), for example, glycolaldehyde and acrolein. Importantly, most biological damages caused by RCS are related to *α*-dicarbonyls (compounds with two adjacent reactive carbonyl groups), including glyoxal, methylglyoxal, 3-deoxyglucosone, 3-deoxyfructose. This is likely because these compounds are about 20,000-fold more reactive than fructose and other reducing carbohydrates (31, 34, 35).

**Figure 3:**
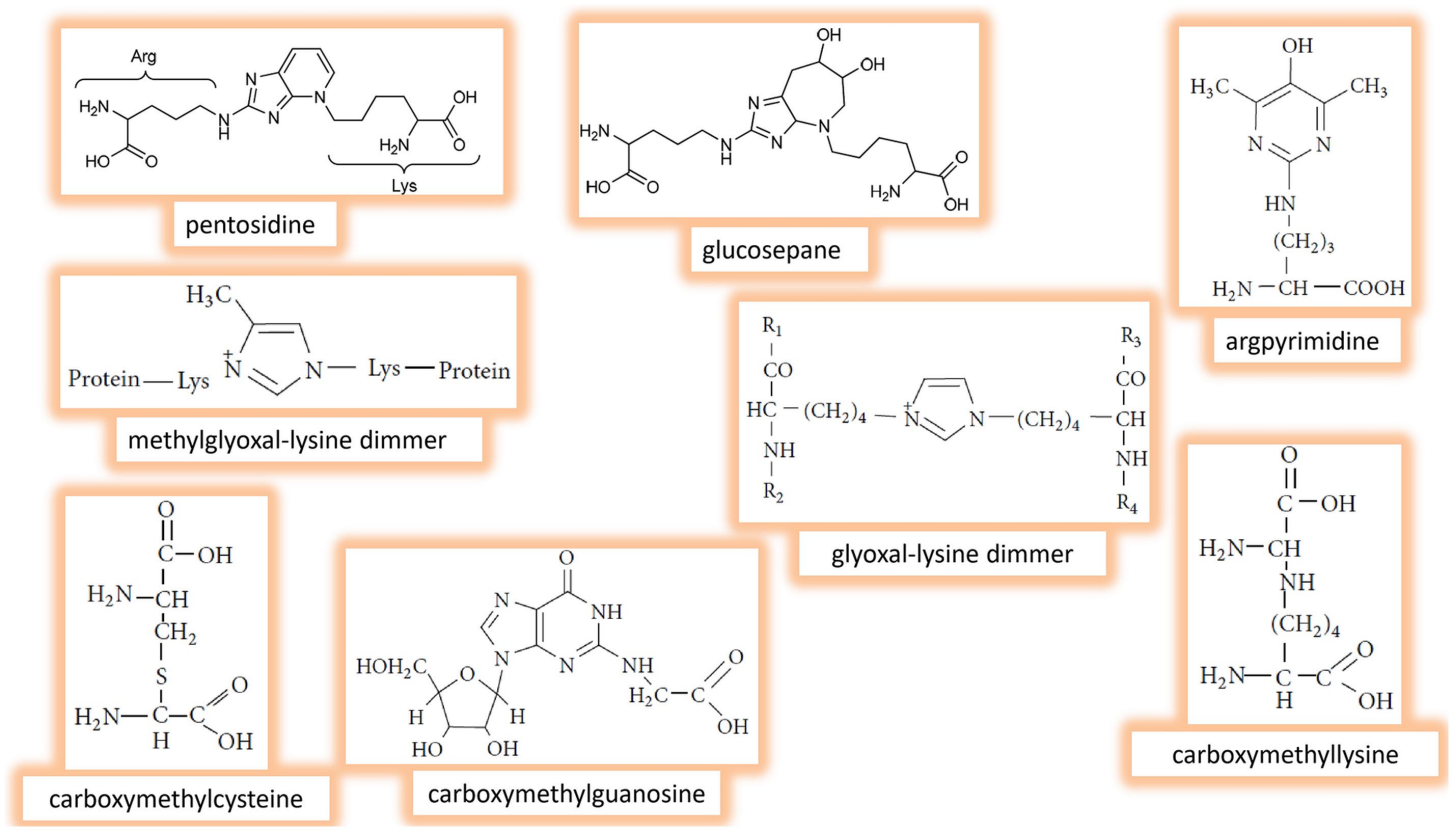
Chemical structure of the main fructose-derived Advanced glycation end products (AGEs) identified *in vivo.*

In the advanced stages of glycation, early products of the process and reactive species further undergo complex of parallel, sequential, and branched chains of irreversible nonenzymatic reactions, ultimately producing a variety of AGEs. Fructose-derived AGEs have been found in various tissues and peripheral blood. Common AGEs detected *in vivo* include pentosidine, glucosepane, argpyrimidine, methylglyoxal-lysine dimmer, glyoxal-lysine dimmer, carboxymethylcysteine, carboxymethylguanosine, carboxymethyllysine, glycolaldehyde-pyridine, 3-deoxyglucosone-lysine dimer, imidizalone, pyrraline, etc. (23, 25, 26). The chemical structures of some AGEs are presented in Figure 3. Generally, AGEs are highly heterogeneous group of compounds with different structures, sizes, and molecular weights. They exhibit various physico-chemical properties; for example, some of AGEs, like pentosidine, are fluorescent, while others, such as methylglyoxal- and glyoxal-lysine dimmers, are nonfluorescent. Also, AGEs may demonstrate different cross-linking abilities (14, 36).

Formation of glycation products is a relatively slow process. In contrast to rapid generation of products in free-radical chain oxidation, AGE formation takes weeks, months, or even years. This could be explained by differences in average reactivities of RCS and ROS and their half-life times. While half-lives of many RCS range from minutes to hours, the most reactive ROS have half-life periods from 10^−9^ to 10^−6^ s (37–39). The slow and irreversible modification of biomolecules via glycation correlates with aging. A causative role of glycation in the aging process and age-associated pathologies has been suggested (40). As confirmation, AGE-modified albumin has been reported to be specifically recognized and degraded by macrophages *in vitro*. Therefore, AGEs have been supposed to serve as specific signals for the recognition and degradation of senescent macromolecules *in vivo*. On the other hand, incomplete removal of glycated proteins by macrophages was connected with aging (41). In fact, the free-radical theory of aging by Harman (42) and the glycation hypothesis of aging by Monnier and Cerami (40) are currently the most popular and widely accepted basis to explain molecular mechanisms of aging.

During glycation, the appearance of reactive species is an unavoidable step toward the formation of AGEs. In addition to this “main direction” (Figure 1, blue arrows), reactive carbonyls can return to the starting point and initiate new rounds of glycation, since RCS have at least one carbonyl group to interact with new biomolecules. It should be noted that most biomolecules are nucleophiles, as many of them, including proteins, amino lipids, nucleic acids, possess amino, sulfhydryl, guanidine or other nucleophilic functional groups. Thus, many different biomolecules are involved in the irreversible formation of AGEs. The latters, in turn generate both RCS and ROS (33), leading to new stages of glycoxidation, creating more and more vicious nonenzymatic chemical cycles (Figure 1, red arrows).

In addition to nonenzymatic processes, normal enzymatic metabolism can also produce reactive species, including RCS. Under physiological conditions, up to 0.4% of glycolytic intermediates can be converted to methylglyoxal, a side product of glycolysis (43). Under other circumstances, reactive carbonyls are generated enzymatically as necessary products. In activated neutrophils, for example, some amino acids are found to be oxidized to different RCS, that demonstrates particular importance of reactive carbonyls in the immune response (44–46). Also, enzymatically produced RCS are involved in regulating gene expression and signaling networks (5, 7, 39). It is important to note that reactive species play a beneficial role only at their low steady-state concentrations, which are tightly controlled by appropriate enzymes in the organism.

Nonetheless, some enzymatic pathways can intensify RCS production to relatively high levels, when reactive carbonyls demonstrate detrimental effects. For example, the polyol pathway, which is responsible for converting glucose to fructose, increases under hyperglycemia in the insulin-insensitive tissues. As mentioned, fructose itself may initiate glycation and be converted to RCS. In both humans and animal models, increased fructose and enhanced flux of polyols have been suggested as metabolic basis for different pathologies (47, 48).

Importantly, high fructose diet, as a source of exogenous fructose, is clearly related to deleterious side effects, including metabolic syndrome, kidney and heart diseases, insulin resistance and type 2 diabetes, nonalcoholic fatty liver disease, arthritis, dementia and neurodegenerative disorders. Glycation is believed to contribute significantly to the fructose-mediated pathogenesis. As confirmation, chronic exposure to high exogenous fructose has been observed to stimulate RCS/ROS formation and AGE accumulation in various *in vitro* and *in vivo* experimental models (17, 22, 32, 49–51). Moreover, excessive fructose and glycation products can alter gut microbiota composition, potentially leading to inflammatory events. Overconsumption of fructose (above 50 g per day) is one of the major factors contributing to chronic inflammation and related diseases (20, 52).

Conversely, epidemiological, clinical, and experimental studies suggest that fructose intake of less than 50 g per day is a moderate dose that may have no detrimental influence (53–56). Moreover, clinical investigations evidenced that small doses of fructose (≤10 g/meal) significantly decreased the postprandial glycemic response to high glycemic index foods and demonstrated beneficial effect on the levels of HbA1c and triglycerides, as well as improved blood pressure and body mass index (53, 57–60). In addition, the Dietary Guidelines for Americans recommend limiting calories from added sugars to less than 10% of total daily calories (61). This recommendation corresponds well to the mentioned above moderate doses of fructose.

According to Marriott and colleagues (62), the highest mean percentage of added fructose intake is associated to the consumption of nonalcoholic beverages (54.3%), grain products (20.3%), and sweets (12.1%). Also, the highest mean percentage of total fructose consumption is linked to nonalcoholic beverages (46.0%), grain products (17.3%), fruits and fruit products (13.4%), sweets (10.3%), milk and milk products (7.1%), and vegetables and vegetable products (2.7%).

Most foods contain reactive carbonyls and AGEs, in fact, in substantive amounts as compared to their endogenous sources, making them a major exogenous source of glycation products, known as dietary glycotoxins (61–67). For example, the amount of ingested Heyns products (calculated as fructoselysine) per day ranges between 500 and 1,200 mg, while the quantity of dietary AGEs (mainly pyrraline and carboxymethyllysine) ranges from 25 to 75 mg (68). High-fructose corn syrup, a widely used sweetener in many foods and beverages, is the major source of such RCS as 3-deoxyglucosone, glyoxal, and methylglyoxal (69–71). Consequently, the Western diet, which is rich in processed and sweetened foods, is strongly associated with a high intake of dietary glycotoxins.

Several comprehensive works demonstrate the content of AGEs in commonly consumed foods, providing a solid foundation for creating dietary AGE database (67, 72–74). Unfortunately, there are no standardized conventional methods for measuring dietary AGEs and other glycotoxins, which limits inter-laboratory comparisons of the data obtained. Nevertheless, the AGE levels in common foods are examined and some studies report how AGE content varies by food category and cooking method. It has been shown that total amounts of AGEs were high in processed nuts, bakery products, and certain types of meats and cereals (>150 mg/kg), while AGE content is rather low in dairy products, vegetables, and fruits (< 40 mg/kg) (73).

In fact, AGEs are unavoidable components of our daily meals, as they are present in natural, uncooked foods. However, processed foods—especially those high in fat, sugar, and protein (Western diet)—tend to be particularly rich in AGEs. Any food processing increases AGE content in regime-dependent manner during cooking. It has been well demonstrated that frying, broiling, grilling, and roasting substantially accelerated dietary AGE formation, whereas foods cooked by steaming, stewing, poaching, or boiling had relatively low amounts of AGEs. For example, grilled, boiled, and raw carrots had approximately 220, 25, and 10 AGE kU/100 g, respectively. At the same time, fried, poached, and raw chicken contained about 7,000, 1,000, and 800 AGE kU/100 g (67).

Consumption of AGE-rich diet, in which the amounts of AGEs were 3-fold higher than in a regular diet, has been shown to elevate blood concentration of AGEs by about 1.5-fold (75). It has been also observed that up to 30% of dietary AGEs were absorbed in human organism, two-thirds of the absorbed AGEs remained in the body and one-third was excreted during the next 3 days (75–77). Interestingly, the comparison of AGE concentrations in plasma of vegetarian and omnivorous individuals presented significantly higher (by 25%) AGE levels in vegetarians. In comparison with the omnivores, vegans, lacto-ovo-vegetarians, and semi-vegetarians demonstrated the levels of some AGEs higher by 15%, 32%, and 22%, respectively (18, 78).

As noted, some biological effects of AGEs result from their involvement in generating RCS/ROS (Figure 1). An increase in the levels of reactive species leads, in turn, to chronic carbonyl/oxidative stress development and modification of biomolecules with consequent loss of their functions. Also, ingested AGEs elicit toxicological effects by disrupting gut microbiome and immune homeostasis (79, 80). Both exogenously and endogenously derived AGEs are responsible for modulating certain signaling pathways. In particular, they bind to RAGE (Figure 1) and activate the RAGE-downstream pathway, which is involved in a variety of biological processes from proliferation and inflammation to apoptosis and autophagy (15, 20, 81). Upregulation of RAGE is known mostly for playing a detrimental role associated with various pathologies.

## Multifaceted RAGE

The Receptor for AGEs (RAGE) is a member of the immunoglobulin superfamily of cell-surface molecules. Originally purified from bovine lung endothelial cells (82, 83), RAGE is now known to be an ubiquitous receptor present in a variety of both immune and nonimmune cells (leucocytes, monocytes, macrophages, pericytes, and endothelial cells, etc.). RAGE is found in various organs and tissues such as the heart, liver, lungs, kidneys, skeletal muscles, and, the cardiovascular and nervous systems (84). Besides AGEs, RAGE recognizes a number of other ligands: (1) damage-associated molecular pattern molecules (DAMPs)-amyloid-*β* peptides (Aβ), β-sheet fibrils, high mobility group box 1 (HMGB1), and S100/calgranulinprotein; (2) pathogen-associated molecular pattern (PAMP) lipopolysaccharide (LPS); and (3) DAMP/PAMP DNA and RNA species (15, 85, 86). The above demonstrates that RAGE is the multiligand receptor, which is able to bind various compounds with different structures, sizes, functions, stimulating thereby multiple downstream signaling cascades.

**Figure 4:**
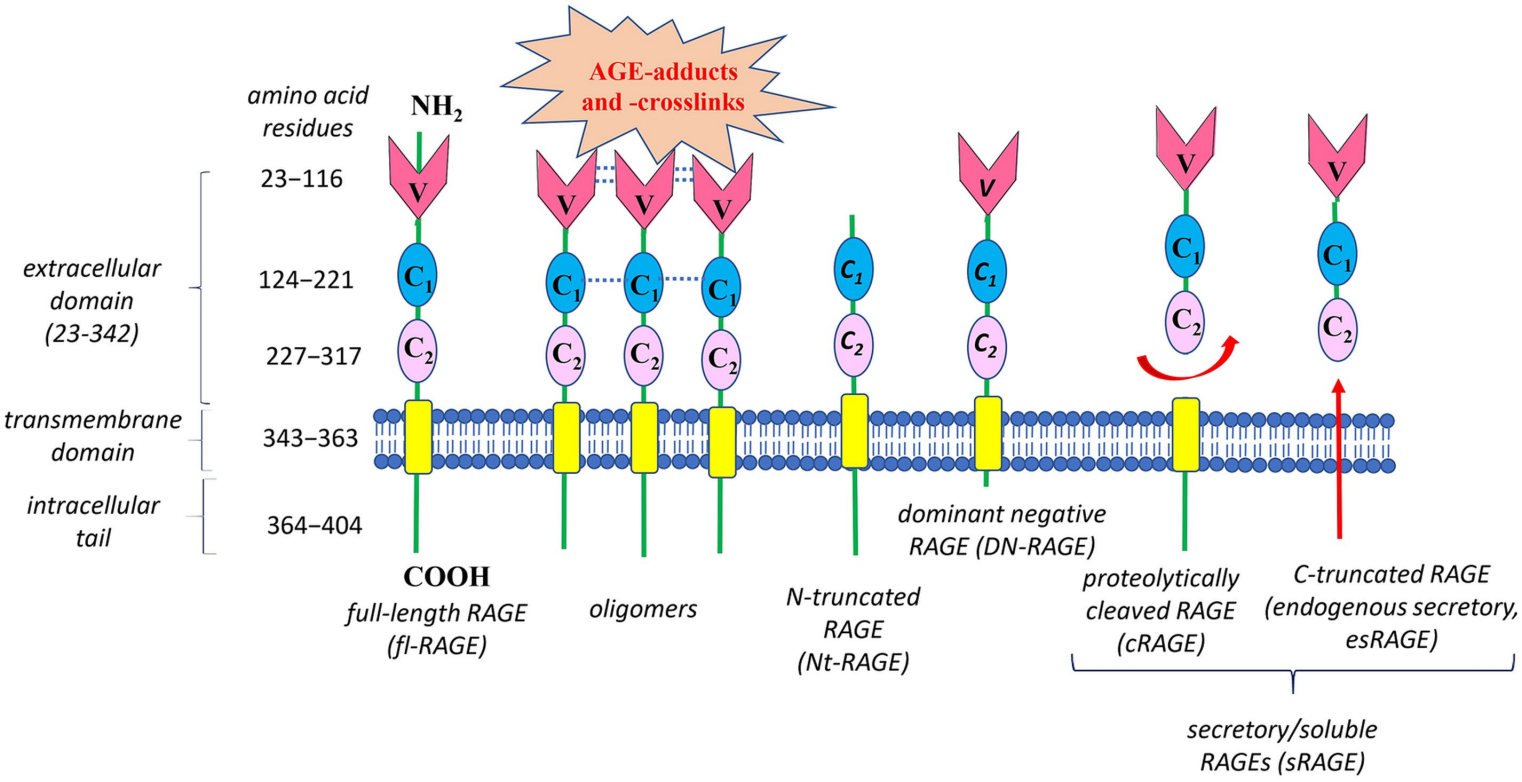
Structure of the human Receptor for advanced glycation end products (RAGE) and its main isoforms. Human RAGE has three domains: (1) an extracellular part responsible for ligand binding, consisting of three immunoglobulin-like parts – a variable N-terminal V-type domain followed by two constant C1- and C2-type domains; (2) a hydrophobic transmembrane region responsible for anchoring on the membrane; and (3) a cytoplasmic tail required for RAGE ligand-mediated signal transduction. Oligomerization of RAGE and its ligands is an important mechanism for controlling AGE-RAGE interplay. Oligomeric ligands can initiate self-association of the V-V/C1-C1 regions in RAGE, forming stable VC1-ligand complex with increased affinity to RAGE ligands, in particular AGE-adducts and AGE-crosslink. The best-studied RAGE isoforms are full-length RAGE (fl-RAGE), N-truncated RAGE (Nt-RAGE), dominant negative RAGE (DN-RAGE), and two soluble/secretory forms (sRAGE)—proteolytically cleaved RAGE (cRAGE) and C-truncated RAGE (endogenous secretory RAGE, esRAGE). Deletion of the V-type domain transforms fl-RAGE into the Nt-RAGE variant, which is a membrane-bound protein like fl-RAGE, but lacking the ability to bind AGEs. The DN-RAGE form has the extracellular (V- and C-type regions) and transmembrane domains like fl-RAGE, but lacks the intracellular cytoplasmic domain required for initiating downstream signaling. Circulating sRAGE has no the intracellular signaling tail and transmembrane domain. sRAGE is a heterogeneous group of proteins resulting from either proteolytic cleavage of fl-RAGE from the cell surface (cRAGE) or alternative splicing of fl-RAGE mRNA (esRAGE).

What molecular mechanisms enable RAGE to recognize such a wide range of ligands and contribute to its diverse bioeffects? Structural studies reveal that full-length human RAGE has three domains (Figure 4): an extracellular part responsible for ligand binding, a hydrophobic transmembrane spanning region, and a highly charged cytoplasmic tail, which is essential for RAGE ligand-mediated signal transduction (83, 87).

The extracellular fragment of RAGE consists of three immunoglobulin-like parts: a variable N-terminal V-type domain, followed by two constant C1- and C2-type domains. The V-type domain is essential for ligand binding. Interestingly, both *in vitro* and *in vivo* studies report that V-type and the adjacent C1-type domains are able to form structural and functional VC1 tandem unit with multiple binding sites to recognize numerous different RAGE ligands (87–89). These ligands interact with positively charged VC1 complex in a heterogeneous manner and, at first sight, they have no significant structural similarity. Nevertheless, a diverse range of RAGE ligands have at least one common feature – their acidic nature (negative charge) (90), though hydrophobicity-dependent mechanisms for RAGE-ligands interaction are also important (91).

In addition to the mentioned above, oligomerization of either RAGE or its ligands is one more key mechanism, controlling AGE-RAGE interplay (90). For example, oligomeric ligands can initiate self-association of the V-V/C1-C1regions in RAGE (Figure 4), forming relatively stable VC1-ligand complex with increased affinity to a diverse spectrum of ligands, including AGE-adducts and AGE-crosslinks. Also, such clustering shifts the equilibrium between monomer/oligomeric states of RAGE and respective ligands. This creates a positive feed-back loop and amplifies the RAGE ligand-mediated signal transduction. Under normal physiological conditions, RAGE expression is rather low. In contrast, during chronic inflammation RAGE is upregulated due to elevation of its ligand levels and the amplifying mechanisms of RAGE activation (92, 93). Generally, increased levels of AGEs, along with oligomerization and activation of RAGE, may explain why the RAGE signaling pathway is highly activated in various inflammation-related pathologies.

Obviously, the oligomerization is not the only mechanism underlying AGE-RAGE axis operation. Due to alternative splicing and metalloprotease-mediated cleavage, several variants of human RAGE appear, which regulate downstream pathways (84, 93). Figure 4 demonstrates the structures of the best-characterized RAGE isoforms: full-length RAGE (fl-RAGE), N-truncated RAGE (Nt-RAGE), dominant negative RAGE (DN-RAGE), and two soluble/secretory forms (sRAGE)—proteolytically cleaved RAGE (cRAGE) and C-truncated RAGE (endogenous secretory, esRAGE).

Deletion of the V-type domain transforms fl-RAGE into the Nt-RAGE variant, which is membrane-bound protein like fl-RAGE, but lacking the ability to bind AGEs. The Nt-RAGE protein is suggested to be involved in the control of angiogenesis by regulating AGE-independent migration of endothelial cells (94). Overexpression of Nt-RAGE may contribute to pathological events independently on the V-type domain.

The DN-RAGE form has the extracellular (V- and C-type regions) and transmembrane domains like fl-RAGE, but lacks the intracellular cytoplasmic domain required for initiating downstream signaling. Consequently, DN-RAGE binds AGE ligands, forming complexes that are not signaling-competent. Moreover, DN-RAGE can suppress RAGE-induced signaling by clustering with fl-RAGE and formation of hetero-complexes able to bind extracellular ligands without transducing signals into the cell. It is highly possible, that under certain conditions, DN-RAGE formation serves as an important mechanism to attenuate of fl-RAGE-induced signaling. For example, DN-RAGE has been found to inhibit tumor cell proliferation and invasion *in vitro* and tumorigenesis *in vivo* (95). Under normal physiological conditions, the level of DN-RAGE expression is similar to that of fl-RAGE (96).

Similar to DN-RAGE, sRAGE has no intracellular signaling tail, but also does not have the transmembrane domain, thus it is circulating protein. sRAGE is a heterogeneous group of proteins, resulting from either alternative splicing of fl-RAGE mRNA (esRAGE) or proteolytic cleavage of fl-RAGE from the cell surface (cRAGE) (84, 93). A unique 16-amino-acid segment in the C2 domain distinguishes esRAGE from cRAGE. The serum level of cRAGE is 5-fold higher than that of esRAGE in healthy individuals (97). Both sRAGE forms are believed to be competitive inhibitors of RAGE. By possessing the same AGE-binding ability as fl-RAGE, sRAGE acts as a “decoy” receptor, preventing circulating AGEs from interacting with RAGE (98), and thus providing cytoprotective effects against the AGE-RAGE complex negative impact. In addition, similar to DN-RAGE, esRAGE can interact with fl-RAGE, leading to the formation of hetero-complexes, which can bind extracellular ligands, but without transducing a signal into the cell.

Overall, understanding how various fructose-derived AGEs interact with multifaceted RAGE opens numerous ways to influence both the normal physiological and pathological events in the organism. Growing evidence supports a dual role of fructose itself, fructose-derived glycation products, and RAGE (21, 23, 93, 99–101). However, the AGE-RAGE axis is primarily known for its adverse effects on cellular metabolism and function through inflammation and nonenzymatic processes. For instance, the AGE-RAGE signaling pathway is believed to induce the expression of proinflammatory modulators (IL-6, TNF-*α*, IFN-*γ*) via activation of the transcription nuclear factor kappa-B (NF-κB) (15). Also, NF-κB increases RAGE expression, which in turn again stimulates NF-κB (88), creating a vicious cycle and generating more and more reactive species in new rounds (Figure 1). Additionally, RAGE expression induces NADPH oxidase-dependent generation of superoxide anion-radical and hydrogen peroxide (15), which contribute to the production of new AGEs as additional RAGE ligands. Overexpression of RAGE has been observed in various pathologies across a wide range of cells, including hepatocytes, cardiomyocytes, neuronal cells, vascular endothelial cells, and renal cells. Upregulation of RAGE was also found in such immune cells as neutrophils, monocytes/macrophages, lymphocytes, dendritic cells, where its activation resulted in the induction of expression of NF-κB-regulated genes encoding proinflammatory cytokines, chemokines, and adhesion molecules (15, 102–104).

## Approaches for mild modulation of the AGE-RAGE axis

**Figure 5:**
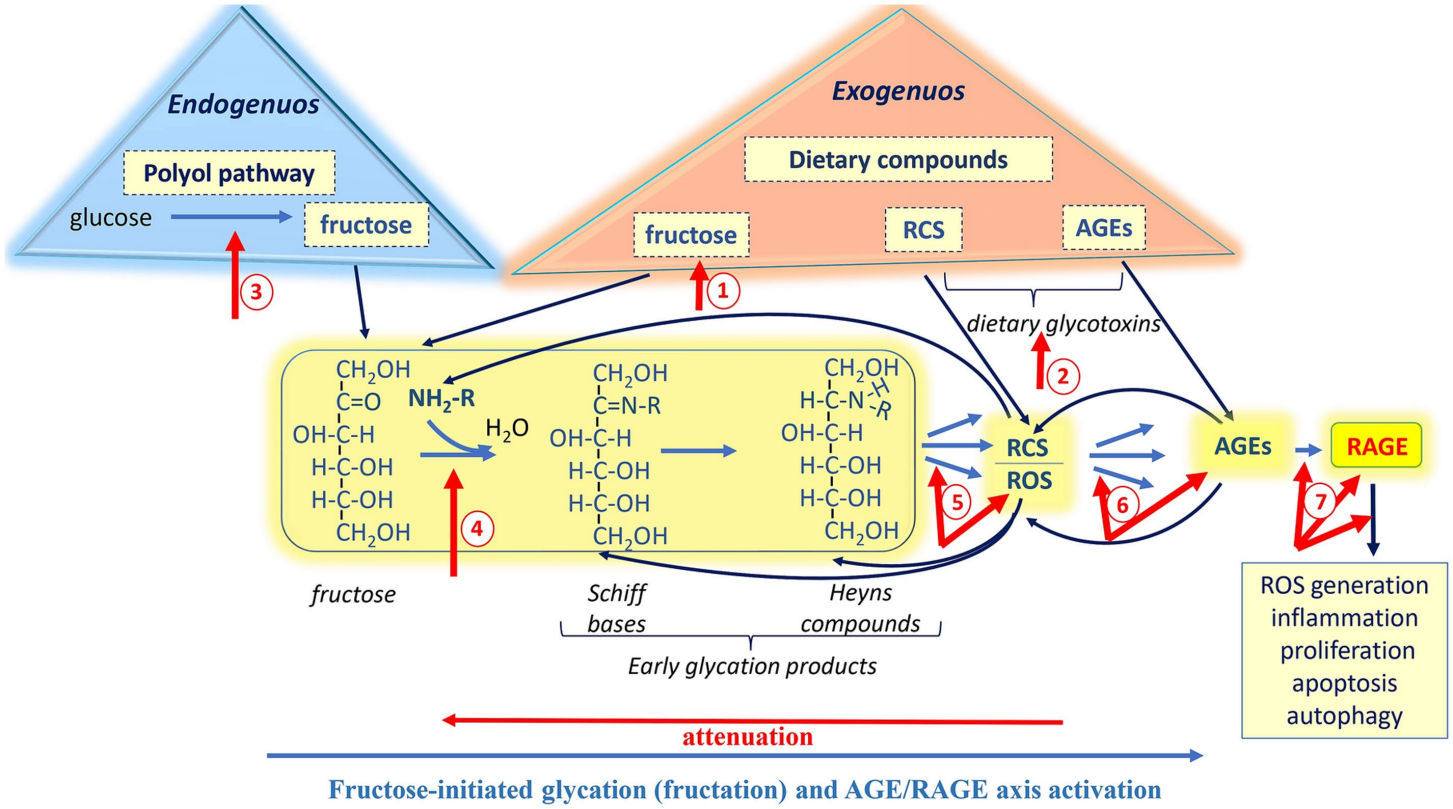
Potential targets іn the pathway from fructose to RAGE for mild modulation of the AGE-RAGE axis. The strategies toward the modulation of fructose-initiated AGE-RAGE axis include: (1) prevention of RCS/AGE formation (red arrows 1–4); (2) reduction of RCS/AGE levels (red arrows 4–6); and (3) suppression of RAGE-mediated signaling (red arrows 7). Mild approaches such as diet optimization, utilization of functional foods, proper intake of probiotics, and regular moderate physical training may provide moderate control of the fructose-mediated AGE-RAGE axis and help manage the adverse effects associated with its overactivation.

At least the aforementioned points suggest that attenuation of the AGE-RAGE signaling could be a beneficial approach to preventing fructose-mediated disturbances. While fructose is a valuable, healthy, and safe nutrient at moderate intake levels (≤50 g/day or < 10% of total calories per day) (53–56), long-term consumption of excessive fructose and elevated endogenous its production have been shown to have detrimental impacts (19–23, 47, 48). There several potential targets within the pathway from fructose to active RAGE that could be considered for modulating the fructose-initiated AGE-RAGE axis (Figure 5, red arrows 1–7). These targets to some extension may serve as “stepping stones” in the following strategies: (1) prevention of RCS/AGE formation, (2) reduction of RCS/AGE levels, and (3) suppression of RAGE-mediated signaling.

Most importantly, the human organism itself possesses a highly developed and tightly regulated antiglycoxidation system that operates at various levels to protect against the adverse effects of AGE-RAGE axis overactivation. Also, it should be kept in mind that strategies to counteract the detrimental impacts of RAGE signaling should preserve beneficial effects of RAGE. In this regard, it makes sense to apply mild approaches to modulate the axis.

### Strategy #1 (prevention of RCS/AGE formation)

It is well documented that limiting added sugars, and fructose in particular (Figure 5, red arrow 1), as well as dietary glycotoxins (RCS and AGEs; Figure 5, red arrow 2) has a beneficial impact on health (72, 105–109). For example, fructose- and glycotoxin-restricted diet increases insulin sensitivity, improves renal failure, and reduces cardiovascular risk. Obviously, fructose, RCS, and AGEs are inevitable constituents of our daily diet, therefore completely avoiding them is practically impossible. Nevertheless, reducing the intake of fructose and glycotoxins seems to be an effective strategy for mitigating the adverse health effects associated with the AGE-RAGE axis.

To decrease carbohydrate consumption and/or lower elevated blood glucose levels, some researchers propose substituting carbohydrates with ketone bodies. The latters are substantial energy substrates and can effectively replace carbohydrate requirements under certain circumstances (110). In fact, the level of ketone bodies increases with the consumption of a high fat/low carbohydrate diet, commonly known as a ketogenic diet. However, at high amounts, fats undergo lipid peroxidation, producing both RCS and ROS. Moreover, at high concentrations, ketones can be damaging due to their reactive carbonyl groups. Therefore, ketogenic diet must be well-balanced and optimized. Under chronic hyperglycemia, excessive glucose enters the polyol pathway, where it can be converted to fructose with the consequent detrimental effects. Thus, it is believed that inhibition of aldose reductase, the key enzyme of the polyol pathway (Figure 5, red arrow 3), may be beneficial (111–113), especially regarding the strategy discussed. Natural inhibitors of aldose reductase have been tested in many studies. Generally, such phytochemicals as alkaloids, phenolic compounds, coumarins seem to be promising (114, 115).

Naturally occurring compounds and their synthetic analogs exhibit good inhibitory activity against glycation and RCS/AGE formation (Figure 5, red arrow 4). Many of them are ubiquitous nutrients, some of which belong to plant or fungal secondary metabolites: polyphenols (e.g., ferulic, gallic and caffeic acids, curcumin, resveratrol), vitamins (e.g., thiamin, pyridoxamine), isothiocyanates (e.g., sulforaphane), alkaloids (e.g., berberine), and statins (35, 116–123). Among compounds with antiglycation activity are peptides such as glutathione and carnosine (32, 124–128). Interestingly, besides antiglycation activity, some compounds are powerful antioxidants and demonstrate inhibitory effects on aldose reductase (129), thereby affecting multiple points in the pathway from fructose to active RAGE.

Additionally, some evidence suggests that optimizing diet, proper intake of probiotics, and moderate physical exercise can be efficient tools to maintain gut microbiome, which has been shown to reduce dietary AGE absorption, modulate AGE levels and sRAGE expression (66, 130–132), and thus have a beneficial impact on human health.

### Strategy #2 (reduction of RCS/AGE levels)

Under normal conditions, the multilevel and complex antiglycation system is effective enough at both preventing the formation (Figure 5, red arrows 4, 5 and 6) and reducing the amount of reactive carbonyls and AGEs (Figure 5, red arrows 5 and 6). The following defensive enzymes found to play a key role in the organism protection against glycoxidation: aldo- and keto-reductases, aldehyde and alcohol dehydrogenases, carbonyl reductases, amadoriases (deglycases), cytochromes P450 family, glutathione reductase, glucose-6-phosphate dehydrogenase, glutathione-S-transferases and glutathione-dependent glyoxalase system (2, 14, 133, 134). In fact, many of these enzymes are associated with important antiglycation agents such as reduced glutathione and reduced equivalents NADPH or NADH. Thus, the defensive system includes both high- and low-molecular-weight components. It is worth noting that many of the antiglycation enzymes, along with their low-molecular-weight “assistants,” also belong to the antioxidant system. This again highlights the interplay between glycation and nonenzymatic oxidation.

Common factors and pathways regulating defensive enzymes also demonstrate a close relationship between carbonyl and oxidative stresses. For instance, sulforaphane effectively prevents AGE formation through the Nrf2 activation that in turn induces glyoxalase 1, the key enzyme of glyoxalase system that detoxifies *α*-dicarbonyl compounds (135–137). Besides glyoxalase 1, among numerous targets of the Nrf2 transcription factor are both antioxidant and antiglycation enzymes: superoxide dismutase, catalase, glutathione peroxidase, peroxiredoxin, thioredoxin reductase, 𝛾-glutamylcysteine synthase, glutathione reductase, thioredoxine reductase, glutathione S-transferases, glutathione reductase (2, 8). In addition, sulforaphane has recently been found to modulate immune response by inhibiting the NF-κB and mitogen-activated protein kinase (MAPK) signaling pathways (138). Similar to sulforaphane, resveratrol, ellagitannins, oleanolic and ascorbic acids also protect cells and tissues against damaging by either oxidants or glycation agents through Nrf2-depending signaling (104, 139–141).

It is well demonstrated that widely used biguanide drug metformin, known for reducing blood glucose levels, also demonstrates the inhibitory effect at the early, intermediate, and late stages of glycation, and induces glyoxalase I activity, thereby reducing the level of *α*-dicarbonyl compounds and AGEs (120, 142, 143). *In vitro* and *in vivo* experiments have demonstrated that metformin reversed methylglyoxal-induced downregulation of Nrf2, and therefore prevented methylglyoxal-initiated damage, apoptosis, and inflammation (144).

Reduced glutathione, carnosine, and aminoguanidine also act as scavengers of α-dicarbonyls and other RCS (32, 117, 128). In addition, glutathione and carnosine are well-known antioxidants that prevent the formation of advanced products of nonenzymatic oxidation and glycation (32, 125–128).

Together with the mentioned above defensive mechanisms, lysosomal and proteasomal systems are also important (15, 111), since to some extent, they are involved in the clearance of poorly degraded AGEs (Figure 5, red arrows 6). Unfortunately, both protective systems decrease with age and under pathologies. Elevated nonenzymatic oxidation and/or glycation can inhibit proteolytic activity of the proteasome pathway due to the accumulation of damaged proteins that inhibit proteasomes (133, 145). Interestingly, some studies demonstrate a positive correlation between regular moderate exercise and activity of glyoxalase 1, as well as lysosomal and proteasomal systems (72, 146).

### Strategy #3 (suppression of RAGE-mediated signaling)

Inhibition of RAGE could also be applied toward the modulation of AGE-RAGE axis (Figure 5, red arrows 7). Regarding this strategy, using inhibitors of either RAGE or its downstream targets, as well as RAGE antagonists, appear to be the most effective approaches.

Various compounds have been tested both *in vitro* and *in vivo* to block the AGE binding sites of RAGE and to act as competitive inhibitors of RAGE interaction with intracellular effectors nessesary for signal transduction (91, 147, 148). Many of synthetic substances demonstrate effective inhibition of RAGE-dependent signaling and consequent molecular events. Nonetheless, supporters of mild approaches prompt to modulate AGE-RAGE pathway by using natural compounds instead of artificial inhibitors, as the latter may cause side effects.

An increasing number of recent studies demonstrate that bioactive compounds derived from different natural sources effectively block AGE-RAGE formation and reduce RAGE signaling (119, 149, 150). For example, scalarin, a marine product, inhibits RAGE (151) and apigenin, a natural flavone, downregulates RAGE/NF-κB expression (152). The mentioned above food-derived scavengers of RCS/AGEs, and in particular sulforaphane, resveratrol, and curcumin, can also downregulate RAGE expression. Sulforaphane reduces expression of RAGE and effectively suppresses the RAGE-depending JNK and MAPK signaling cascades, which in turn inhibits NF-κB pathway and the production of pro-inflammatory cytokines (35, 136, 138, 153, 154).

Interestingly, a recent study reported that ascorbic acid significantly reduced the level of circulating HMGB1 (high mobility group box 1), a ligand of RAGE (141). Similarly, oleanolic acid demonstrated an inhibitory effect on HMGB1 release (139). Therefore, we suggest that such ubiquitous compounds as ascorbic and oleanolic acids also lead to mild reduction of RAGE activity by decreasing the concentration of ligands for RAGE other than AGEs. Moreover, as effective antioxidants, both acids can break glycation-oxidation vicious cycle that leads to AGE formation. In this regard, compounds such as sulforaphane, as well as ascorbic and oleanolic acids, that can control RAGE-mediated events at various points in the glycation-RAGE network, are valuable and promising bioactive components of functional foods for protecting against inflammation, aging and related disorders.

Physical training is another attractive approach for moderate modulation of AGE-RAGE axis. Indeed, in animals studies, exercise has been shown to attenuate the impact of glycation by elevated activity of glyoxalase 1 with simultaneous reduced activity of aldose reductase and decreased total RCS/AGE levels (155). Also, rergular moderate exercise has been reported to decrease the expression of the RAGE/NF-κB pathway (156). However, human studies often demonstrate contradictory results. As observed, the expression of RAGE, and sRAGE in particular, can vary significantly depending very much on the type and intensity of physical activity as well as the physiological state of the individuals.

Growing body of research reveals that circulating soluble sRAGE acts as “decoy” for RAGE ligands and can bind to AGEs but does not initiate intracellular signal transduction cascade; thereby upregulation of sRAGE is suggested to elicit beneficial protective effects via reduction of the AGE-RAGE interaction. Animal studies have shown that increasing sRAGE levels can prevent RAGE-mediated dysfunctions and even reverse some detrimental RAGE-related consequences (157–161). These findings confirm that elevating sRAGE could be an effective approach toward the modulating immune system and treating inflammatory diseases. In this regard, numerous studies look for relationship between human lifestyle improvement and sRAGE increase.

However, the impact of physical activity on sRAGE levels is complicated. Some studies reported significant increases in serum sRAGE levels due to physical training, while others found no effect or even a decrease in sRAGE levels under certain conditions (132, 162–164). Combinational approach to improve diet and optimize physical activity also sometimes may have unpredictable effects on metabolic parameters (72, 162). Clearly, the effect of physical training on sRAGE levels depends on multiple factors, including the type, duration, and intensity of activity, as well as diet, lifestyle, age, and the individual’s physiological state.

Moreover, sRAGE is a heterogenous group that includes at least the cRAGE and esRAGE isoforms. The mechanisms responsible for the balancing these isoforms in different cases are not well understood. For example, cRAGE and esRAGE amounts differently change, dependently on the intensity of physical activity (163, 164). Interestingly, total sRAGE level, and cRAGE in particular, decline with age, while esRAGE level does not change during aging, suggesting different associations of sRAGE isoforms with age-related diseases (100).

Administration of sRAGE, either peripherally or directly to target organs, is one of the most attractive and intensively investigated approaches. Accumulating evidence has revealed that in animal models sRAGE administration was associated with reduction in the development of pathological conditions and the improvement in tissue functioning (165). However, some reports indicate that sRAGE elevation correlates with inflammatory conditions (166, 167). It is likely that benefits of sRAGE elevation might be offset by its disadvantages or vice versa, depending on the chronic or acute character of disorder, stages of disease, physiological state of organism, etc. More research is needed to fully understand the effects of sRAGE administration.

## Conclusions and perspectives

Over recent decades, the impact of fructose on human health has attracted growing attention from the scientific community. The “fructose hypothesis” suggests that fructose plays a causative role in various health problems. Indeed, compelling evidence indicates that long-term intake of excessive fructose can have deleterious side effects, which are believed to be attributed to glycation and fructose-derived AGEs. It is well established that AGEs are involved in upregulation of the specific receptor for AGEs (RAGE), amplifying RAGE-mediated signaling related to inflammation, metabolic disorders, chronic diseases, and aging. In contrast, short-term consumption of low/moderate doses of fructose are found to be beneficial, as it contributes to decrease of blood pressure, body mass index, the levels of triglycerides and HbA1c, while also improving glucose tolerance. Fructose is a valuable nutrient under certain pathophysiological conditions, particularly due to inducing mild carbonyl/oxidative stress, which in turn stimulates protective mechanisms.

Recent advances in studying the AGE-RAGE axis have significantly extended our understanding of its crucial role in human health. Nevertheless, several key issues remain to be addressed. Unfortunately, there are no standardized conventional methods to measure dietary AGEs and other glycotoxins, which limits inter-laboratory comparisons of the data obtained. This limitation hinders developing a unified dietary AGE/glycotoxin database. Standardized methods also could help determine whether there are differences between fructose-derived AGEs and those generated by other dietary carbohydrates, such as glucose or galactose. Understanding these distinctions may provide insights into the specific characteristics of AGE-RAGE interactions.

Additional intriguing questions arise regarding the beneficial effects of low doses of fructose. Specifically, does this impact involve AGE-RAGE signaling? If so, which RAGE isoforms are responsible for the benefits associated with fructose? Furthermore, is it possible to optimize the RAGE/sRAGE ratio by using appropriate doses of fructose and other natural compounds in our diets?

While the AGE-RAGE axis downregulation appears to be a promising approach for attenuating various disease conditions linked to RAGE-mediated inflammation, it is crucial to ensure that strategies aimed at counteracting RAGE signaling do not diminish its beneficial effects. Although the structure of the AGE-RAGE network is relatively well-studied, further research is needed to expand our understanding of the mechanisms underlying the operation of the AGE-RAGE axis and their relationship to human health.
